# Efficacy and Safety of a Probiotic Containing *Saccharomyces boulardii* CNCM I-745 in the Treatment of Small Intestinal Bacterial Overgrowth in Decompensated Cirrhosis: Randomized, Placebo-Controlled Study

**DOI:** 10.3390/jcm13030919

**Published:** 2024-02-05

**Authors:** Irina Efremova, Roman Maslennikov, Maria Zharkova, Elena Poluektova, Nona Benuni, Aleksandr Kotusov, Tatyana Demina, Aleksandra Ivleva, Farida Adzhieva, Taisiya Krylova, Vladimir Ivashkin

**Affiliations:** 1 Department of Internal Medicine, Gastroenterology and Hepatology, Sechenov University, Moscow 119992, Russiazharkovamaria@mail.ru (M.Z.); polouektova@rambler.ru (E.P.); nona.benuni@rambler.ru (N.B.); alexanderkotusov@gmail.com (A.K.); la.ivva@yandex.ru (A.I.); krylovataisia@gmail.com (T.K.);; 2 The Interregional Public Organization “Scientific Community for the Promotion of the Clinical Study of the Human Microbiome”, Moscow 119435, Russia

**Keywords:** probiotics, hemodynamics, gut–liver axis, gut–heart axis, vasodilatation, endotoxemia, liver, microbiota, leaky gut

## Abstract

(1) **Background**: The aim was to evaluate the effectiveness of the probiotic containing *Saccharomyces boulardii* in the treatment of small intestinal bacterial overgrowth (SIBO) in patients with decompensated cirrhosis. (2) **Methods**: This was a blinded, randomized, placebo-controlled study. (3) **Results**: After 3 months of treatment, SIBO was absent in 80.0% of patients in the probiotic group and in 23.1% of patients in the placebo group (*p* = 0.002). The patients with eliminated SIBO had decreased frequency of ascites and hepatic encephalopathy, the increased platelets and albumin levels, the decreased blood levels of total bilirubin, biomarkers of bacterial translocation (lipopolysaccharide [LPS]) and systemic inflammation (C-reactive protein), and positive changes in markers of hyperdynamic circulation compared with the state at inclusion. There were no significant changes in the claudin 3 level (the intestinal barrier biomarker) in these patients. No significant changes were observed in the group of patients with persistent SIBO. The serum level of nitrate (endothelial dysfunction biomarker) was lower in patients with eradicated SIBO than in patients with persistent SIBO. One (5.3%) patient with eradicated SIBO and six (42.9%) patients with persistent SIBO died within the first year of follow-up (*p* = 0.007). (4) **Conclusions**: SIBO eradication was an independent predictor of a favorable prognosis during the first year of follow-up.

## 1. Introduction

Cirrhosis is the final stage of chronic liver damage [1]. According to a recent review, there are more than 100 million people with cirrhosis worldwide [2,3,4]. This disease is responsible for a significant number of premature deaths and disabilities [2,3,4]. Small intestinal bacterial overgrowth (SIBO) is an increase in the number of bacteria in the small intestine to more than 100,000 cells per mL of contents [5,6,7,8]. According to a number studies and their meta-analysis, SIBO in decompensated cirrhosis is detected in more than half of patients, and its presence is associated with ascites, systemic inflammation, hemodynamic changes and an unfavorable prognosis [9,10,11]. The main treatment for SIBO is taking antibacterial medications [8]. However, this is associated with the development of antibacterial resistance, changes in the gut microbiota composition and the development of antibiotic-associated gastrointestinal lesions [12,13,14,15,16,17]. In this regard, an important task of modern gastroenterology is the search for alternative drugs for the eradication of SIBO. Among these candidates, probiotics are among the most promising. Probiotics have a complex effect on the gastrointestinal tract and its microbiota, including selectively suppressing the growth of certain types of pathogenic and conditionally pathogenic microbiota [18,19,20,21,22]. Among the probiotic microbes of interest are *Saccharomyces boulardii*, which have already shown an antagonistic effect against *Helicobacter pylori* [23] and *Clostridioides difficile* [24,25]. However, they have not yet been tested for the treatment of SIBO, including in cirrhosis. The aim of our study was to evaluate the effectiveness and safety of a probiotic containing *Saccharomyces boulardii* in the treatment of SIBO in patients with decompensated cirrhosis.

## 2. Materials and Methods

This randomized, single-blind, placebo-controlled study was approved by the local ethics committee of Sechenov University (protocol #22-21 dated 9 December 21) in accordance with the Declaration of Helsinki. All included patients signed informed consent to participate in the study. This study was part of a larger study registered at clinicaltrials.gov (NCT05231772).

### 2.1. Patients

The inclusion criteria were as follows: cirrhosis diagnosed on the basis of histopathological or clinical, biochemical and ultrasonographic data; Child–Pugh class B or C cirrhosis; age from 18 to 70 years; signed informed consent; and presence of SIBO. The exclusion criteria were as follows: intake of prebiotics, probiotics, antibiotics, or metformin within 6 weeks prior the inclusion; alcohol consumption within 6 weeks prior to the inclusion; inflammatory bowel disease, cancer, or any other serious illness. Patients who prematurely discontinued the consumption of tested probiotic/placebo, or started taking antibiotics, other probiotics, or prebiotics during the follow-up period, or refused to participate during the follow-up period were also excluded from the study.

Based on the study of Furnari M et al. [26], who tried to eliminate SIBO in 23 patients with cystic fibrosis using rifaximin (13 patients in the rifaximan group, 10 patients in the control group), the required sample size with a power of 0.85 should be 12 patients. There are no data available to calculate the required sample size for cirrhosis and the tested probiotic.

**Figure 1:**
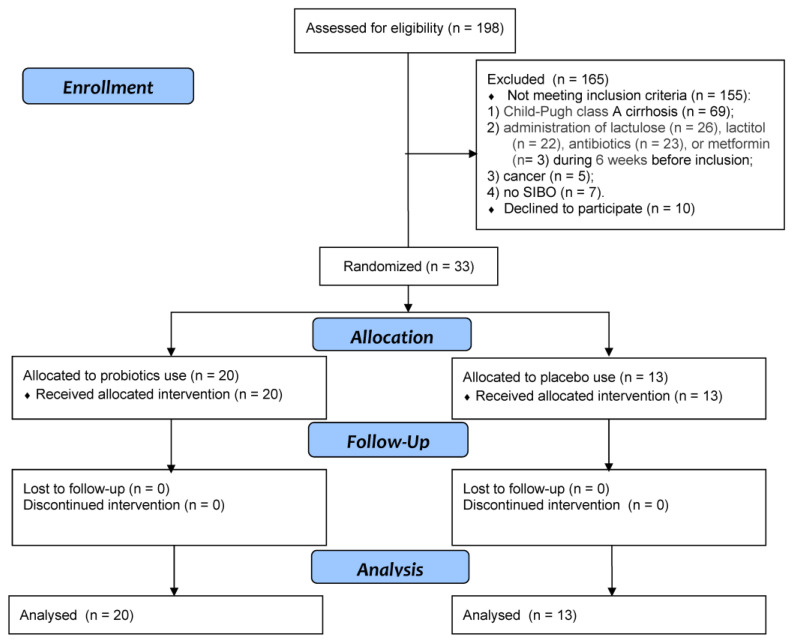
CONSORT 2010 flow diagram.

Of the 198 patients initially screened for inclusion, 33 met the inclusion criteria and were included in the study (Figure 1). Patients included in the study were randomized into test and control groups (ratio 1.5:1). The Excel function RANDBETWEEN (1:5) was used as a random number generator; for numbers 1 to 3, patients were assigned to the probiotic group, and for numbers 4 or 5, patients were assigned to the placebo group.

### 2.2. Intervention and Controls

Patients in the probiotic group received *S. boulardii* CNCM I-745 (Enterol^®^; Biocodex, Gentilly, France) at a dose of 250 mg twice daily for 3 months, and patients in the control group received a placebo at the same dose for the same period. The placebo did not differ in appearance from the tested drug. It had the same composition as the tested drug, except that it did not contain the probiotic, which was replaced by excipients in proportionally increased quantities. The containers that contained the tested drug and placebo also did not differ from each other in appearance. Patients did not know whether they were taking the probiotic or a placebo. In addition, all patients received standard treatment for cirrhosis in accordance with guidelines [27]. Medications taken did not differ significantly between patient groups (Table 1). Due to the presence of cirrhosis, all patients were recommended to completely abstain from alcohol. Patients were re-evaluated 3 months after initiation of *S. boulardii* or placebo treatment.

### 2.3. Outcomes

The primary outcome is the elimination of SIBO at the end of the 3-month treatment period. Secondary outcomes are changes in the severity of cirrhosis manifestations within the 3-month treatment period and the prognosis for the life of patients within the 2-year follow-up period after the end of this treatment period.

At the end of the treatment period, patients returned the containers with drugs/placebo, and compliance was assessed by counting the remaining capsules.

To confirm that the patients were alive, they were contacted every 3 months by phone. We contacted the patient’s relatives on this issue by phone if there was no answer. If we were unable to make contact with them, we studied the patient’s electronic medical record in the Unified Medical Information and Analytical System.

### 2.4. Investigations

At inclusion, each patient underwent a complete blood count, chemical blood test, an international normalized ratio calculation, neurological examination, abdominal ultrasound, esophagogastroduodenoscopy, number connection test to detect minimal hepatic encephalopathy, lactulose hydrogen breath test for SIBO, assessment of the markers of systemic hemodynamics, systemic inflammatory reaction (C-reactive protein [CRP]), bacterial translocation (serum lipopolysaccharide [LPS] [Limulus amebocyte lysate test; photometry; reagent with catalog number EC64405S; Bioendo; Xiamen, China]), intestinal barrier damage (serum claudin 3 [enzyme linked immunosorbent assay; reagent with catalog number SEF293Hu; Cloud-Clone Corp.; Wuhan, China]), and vasodilating endothelial dysfunction (serum nitrates [photometry; reagent with catalog number A013-2; Cloud-Clone Corp.; Wuhan, China]). All of these studies were repeated after 3 months when patients finished taking the probiotic/placebo.

The lactulose hydrogen breath test was used to diagnose SIBO with Gastrolyser (Bedfont, Maidstone, UK) according to North American Consensus [28]. The patient consumed 10 g of lactulose dissolved in 200 mL of water, after which the level of exhaled hydrogen was determined every 15 min for 90 min. Baseline breath hydrogen levels were also measured immediately before lactulose ingestion. We considered SIBO to be present when there was an increase in breath hydrogen of at least 20 ppm above baseline within these 90 min.

To assess systemic hemodynamics, echocardiography in accordance with the guidelines of the American Society of Echocardiography [29,30,31,32] was performed simultaneously with oscillometric determination of systolic and diastolic blood pressure and pulse with a semi-automatic tonometer in the supine position. Left ventricular end-diastolic and end-systolic volumes were determined using the Modified Simpson’s disk method, and ejection fraction was estimated as the ratio of the difference between end-diastolic and end-systolic volumes to end-diastolic volume. Stroke volume was defined as Doppler velocity time integral across the aorta multiplied by the cross-sectional aorta area. Cardiac output was calculated as the product of heart rate and stroke volume [33]. Mean blood pressure was calculated as ([systolic blood pressure] + 2 × [diastolic blood pressure])/3. Systemic vascular resistance was defined as the ratio of mean blood pressure to cardiac output.

If ascites was detected on physical examination, it was considered clinically significant (degrees 2 and 3 according to the International Ascites Club scale). If ascites was detected only on abdominal ultrasound, it was considered minimal (degree 1 according to the International Ascites Club scale).

### 2.5. Statistics

Statistical analysis was performed using STATISTICA 10 software (StatSoft Inc., Tulsa, OK, USA). Data are presented as median [interquartile ranges]. Differences between continuous variables were assessed using the Mann–Whitney test because many variables were not normally distributed. Changes in the values of variables during the study were assessed using the Wilcoxon test. To assess differences between categorical variables, Fisher’s exact test was used. The Kaplan–Meier estimator and the Mantel–Cox test were used for survival assessment. The Cox regression model was used to assess the factors on patient survival and hazard ratio (HR). *p* values ≤ 0.05 were considered statistically significant.

## 3. Results

Patients from the probiotic and placebo groups at the time of inclusion in the study did not differ significantly in gender, age, etiology, manifestations and severity of cirrhosis (Table 1 and Table 2). These patients also did not differ significantly in drug used within the 3 months treatment period (Table 1). All included patients were outpatients.

Directly after the end of the treatment period, all included patients were re-examined. Then, during the 2-year follow-up period, only telephone contact was maintained with them in this study.

**Figure 2:**
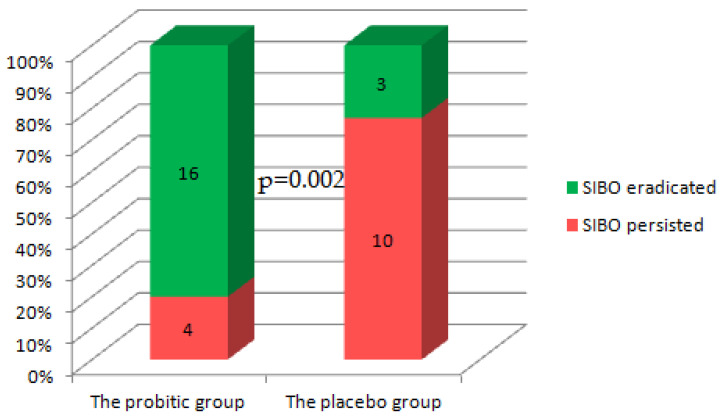
Efficacy of probiotic and placebo in treating small intestinal bacterial overgrowth (SIBO) in patients with decompensated cirrhosis.

After 3 months treatment, SIBO was absent in 80.0% of patients in the probiotic group and in 23.1% of patients in the placebo group (*p* = 0.002; Figure 2). The power of our study was 0.94.

In the probiotic group, as a result of treatment, a significant decrease in the frequency of ascites and hepatic encephalopathy was observed, the blood level of platelets, albumin, sodium and cholesterol increased, and the serum activity of alkaline phosphatase (AP) and aspartate aminotransferase (AST) decreased. In these patients, there was a significant decrease in the severity of cirrhosis according to the Child–Pugh scale, as a result of which 40% of them moved from the group of decompensated cirrhosis (classes B and C) to the group of compensated cirrhosis (class A). In addition, these patients showed a significant decrease in the levels of biomarkers of bacterial translocation (serum LPS) and systemic inflammation (serum CRP), as well as positive changes in markers of hyperdynamic circulation (an increase in systemic vascular resistance and a decrease in left ventricular end-diastolic volume, stroke volume, and cardiac output). These changes were not observed in the placebo group. On the contrary, in that group, there was a decrease in cholinesterase and platelet levels. There was no significant decrease in the level of the intestinal barrier marker claudin 3, international normalized ratio, total bilirubin, alanine aminotransferase in any of the groups, nor were there significant changes in the prevalence of esophageal varices (Table 2).

We compared groups of patients in whom SIBO was eliminated and in whom SIBO persisted, regardless of whether they received a probiotic or a placebo. At inclusion, there were no significant differences between these groups in age, sex distribution, drugs used (except the use of probiotic/placebo), etiology and manifestations of cirrhosis, except the serum total protein level, which was lower due to the lower content of globulins in patients in whom SIBO was subsequently eliminated (Table 3).

The patients with eliminated SIBO had a decreased frequency of ascites and hepatic encephalopathy, increased blood levels of platelets and albumin, decreased blood levels of alkaline phosphatase and total bilirubin, decreased severity of cirrhosis according to the Child–Pugh scale, decreased levels of biomarkers of bacterial translocation and systemic inflammation, as well as positive changes in markers of hyperdynamic circulation (an increase in systemic vascular resistance and a decrease in left ventricular end-diastolic volume, stroke volume, and cardiac output) compared with the state at inclusion. There were no significant changes in the level of the intestinal barrier marker claudin 3, international normalized ratio, and the prevalence of esophageal varices in these patients. In the group of patients with persistent SIBO, no significant changes were observed (Table 3). At the end of treatment, the serum level of nitrate (the biomarker of vasodilating endothelial dysfunction) was lower in patients with eradicated SIBO than in patients with persistent SIBO (82 [24–180] µmol/L; *p* = 0.003).

**Figure 3:**
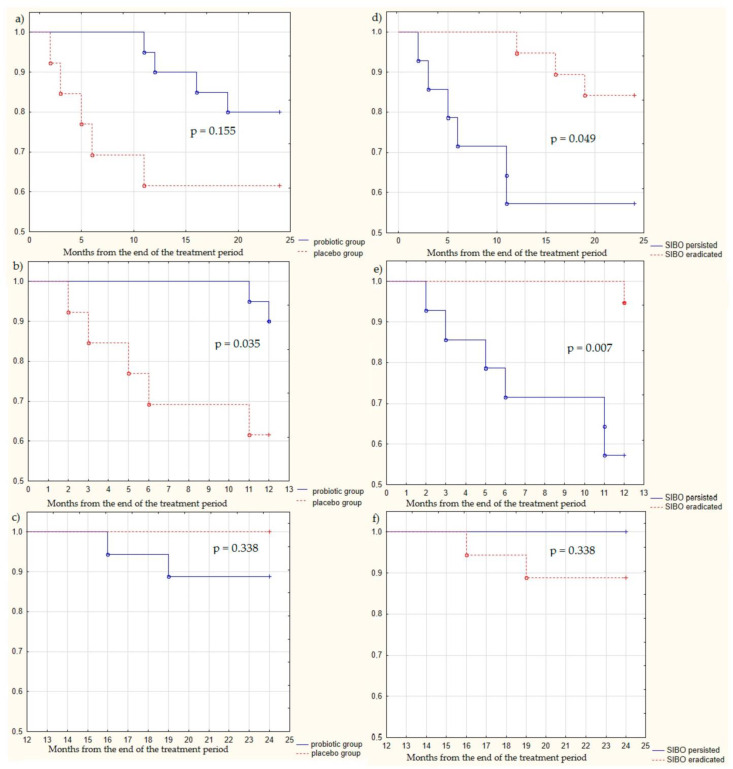
Survival curves of included patients with decompensated cirrhosis: (**a**) in the probiotic and placebo groups throughout the 2-year follow-up period; (**b**) in the probiotic and placebo groups during the first year of follow-up; (**c**) in the probiotic and placebo groups during the second year of follow-up; (**d**) in the groups of eradicated and persistent SIBO throughout the 2-year follow-up; (**e**) in the group of eradicated and persistent SIBO groups during the first year of follow-up; and (**f**) in groups of eradicated and persistent SIBO during the second year of follow-up.

Within 2 years of follow-up period, nine (27.3%) patients died, including four (20.0%) in the probiotic group and five (38.5%) in the placebo group (*p* = 0.155; Figure 3a). Moreover, during the first year of follow-up, 2 (10.0%) patients in the probiotic group and five (38.5%) in the placebo group died (*p* = 0.035; Figure 3b). Among the patients who survived the first year, two (11.1%) from the probiotic group and none in the placebo group died during the second year of follow-up (*p* = 0.338; Figure 3c).

Among patients in whom SIBO was eliminated, three (15.8%) people died within 2 years of follow-up; among those in whom SIBO persisted, six (42.9%) people died within this period (*p* = 0.049; Figure 3d). Moreover, within the first year of follow-up period, one (5.3%) patient died among those in whom SIBO was eradicated by the end of the treatment period, and six (42.9%) patients died among those in whom SIBO persisted (*p* = 0.007; Figure 3e). Among those who survived after 1 year of follow-up, within the second year of follow-up, two (11.1%) patients died in the group wherein SIBO was eradicated by the end of the treatment period, and no patients died in the group wherein SIBO persisted (*p* = 0.338; Figure 3f). Cox multivariate regression analysis showed that eradication of SIBO was an independent predictor of a favorable prognosis for life during the first year of follow-up (Table 4).

Minimal mortality (0% of three cases) was observed in patients with eradicated SIBO in the placebo group. The highest mortality rate (50%) was observed in patients with persistent SIBO in the placebo group, with all deaths occurring within the first year of follow-up. Among patients with eradicated SIBO in the probiotic group, the mortality rate was 18.8%, with all deaths occurring after 11 months of follow-up. Among patients with persistent SIBO in the probiotic group, the mortality rate was 25.0%, with the only person who died dying in the eleventh month of follow-up. Patients in the probiotic group died later than patients in the placebo group (14 [12–18] vs. 5 [3–6] months; *p* = 0.027), and patients with eradicated SIBO died later than patients with persistent SIBO (16 [12–19] vs. 6 [3–11] months; *p* = 0.028). All deceased patients with eradicated SIBO died after 11 months of follow-up, and all deceased patients with persistent SIBO died during the first 11 months of follow-up (*p* = 0.012).

None of the patients developed severe adverse effects from taking the drugs. One patient in the probiotic group developed self-limited itching. 

Compliance in the probiotic and placebo groups was 100%. 

None of the patients consumed alcohol during the treatment period.

## 4. Discussion

Currently, the role of gut microbiota in the progression of cirrhosis has been shown in a number of experimental and clinical studies [34,35,36,37,38,39], so the question of how this target can be managed in the treatment of cirrhosis is actively arising. Disorders of the gut microbiota in cirrhosis were shown to be represented by various pathologies, namely gut dysbiosis and SIBO [40]. If the diagnosis of gut dysbiosis in real clinical practice is still extremely difficult, since methods for assessing it are expensive, rare and not standardized, then SIBO may well be diagnosed in a regular clinic in accordance with clinical guidelines [28]. Therefore, while gut dysbiosis remains a subject of study and debate for scientists, SIBO is already a subject of concern for ordinary doctors.

As mentioned in the Introduction section, numerous studies and their meta-analyses have shown that the presence of SIBO is associated with ascites, hepatic encephalopathy, bacterial translocation, systemic inflammation, vasodilation, and hyperdynamic circulation in cirrhosis [9,10]. All this fits into the concept of the gut–liver–heart axis. SIBO increases the bacterial load on the intestines, which, with increased intestinal permeability characteristic of cirrhosis [41,42,43], leads to bacterial translocation—the penetration of bacteria and their components from the intestinal contents into the intestinal wall, abdominal lymph nodes, ascitic fluid, portal and systemic blood flow. Bacterial translocation causes the development of a systemic inflammation, one of the manifestations of which is NO-mediated systemic vasodilation, causing arterial hypotension. In order to raise the reduced blood pressure, compensatory mechanisms are triggered, leading to fluid retention and an increase in venous return, which is considered a marker of the end-diastolic volume of the left ventricle. The heart pumps an increased volume of blood, which is manifested by an increase in cardiac output. This condition is called hyperdynamic circulation and is characteristic of cirrhosis. The increased amount of blood ejected by the left ventricle of the heart rushes into the abdominal bloodstream, the vessels of which are dilated due to systemic and local inflammation caused by bacterial translocation. This increases blood flow through the portal system, aggravating the course of portal hypertension and increasing the severity of ascites, into which albumin and sodium are lost. In addition, worsening portal hypertension increases blood shunting, which, together with systemic inflammation and increased ammonia production by an increased number of intestinal bacteria, aggravates the course of hepatic encephalopathy [34,36,37]. Long-term persistence of these disorders may explain the worse prognosis of cirrhotic patients with SIBO compared to these patients without SIBO [11].

However, this theory met fair objections that association does not always mean causation. The question remained: what would happen if SIBO was eliminated in cirrhosis patients? Will the liver dysfunctions associated with it and prognosis improve? Only one study has been published on the effect of eliminating SIBO in cirrhosis [44,45]. The non-absorbable antibiotic rifaximin was used as a drug for these purposes. It eliminated SIBO in 76% of cases, and this was accompanied by a decrease in blood ammonia levels and faster performance on psychometric tests for hepatic encephalopathy [45]. The effect of eliminating SIBO on other cirrhosis indicators has not been studied [45], making our study the first to thoroughly examine this issue.

In our study, we showed that eliminating SIBO in cirrhosis has a beneficial effect on the entire gut–liver–heart axis; it reduces the severity of bacterial translocation (whose biomarker was LPS), systemic inflammation (whose biomarker was CRP), inflammatory vasodilating endothelial dysfunction (whose biomarkers were stable NO metabolite nitrates), vasodilation (an increase in systemic vascular resistance), hypervolemia (whose biomarker was the end-diastolic volume of the left ventricle), hyperdynamic circulation, ascites, hypoalbuminemia, and hepatic encephalopathy, which was accompanied by an improved prognosis for the lives of patients. At the same time, it is noteworthy that the positive effect of eliminating SIBO on the prognosis was observed mainly during the first year of follow-up, after which it gradually faded away. The reason for this may be that without eliminating the causes of the development of SIBO in cirrhosis, which are not precisely established, it recurs and again begins to intensify the pathological processes described above, worsening the prognosis.

Interestingly, in our study, neither the use of a probiotic nor the eradication of SIBO had a significant effect on the level of a biomarker of intestinal barrier dysfunction claudin 3 [46] in cirrhosis, which shows that SIBO is likely not associated with this intestinal pathology in cirrhosis; the tested probiotic also did not have a significant effect on the intestinal barrier.

Typically, antibiotics, especially rifaximin, are used to treat SIBO. However, excessive intake of these drugs can contribute to the development of antibacterial resistance and lead to adverse effects. This means an alternative must be found; one such alternative may be probiotics. These drugs have shown their positive effect in the treatment of SIBO in a meta-analysis that summarized data in various diseases [47]. In our study, the effectiveness of the probiotic containing *Saccharomyces boulardii* CNCM I-745 in eradicating SIBO in cirrhosis was 80%, which coincides with the effectiveness of rifaximin (76%) in another study [45]. Further randomized controlled studies directly comparing the effect of rifaximin and probiotics in eliminating SIBO in cirrhosis are required.

Another treatment for SIBO is fecal transplantation, which was shown to be effective in one randomized controlled trial in patients without cirrhosis [48]. Although several studies on the effectiveness of fecal transplantation for cirrhosis have been published [49,50,51], none examined how this procedure affects SIBO, which represents a challenge for future research.

The exact mechanism by Ih *Saccharomyces boulardii* CNCM I-745 eliminates SIBO is not clear. This may be due to antagonism to small intestinal bacteria, the overgrowth of which is SIBO, or to the normalization of small intestinal motility, the slowdown of which is associated with the development of SIBO in cirrhosis [9]. New research is needed to answer this question.

A limitation of this study is the small number of participants, which did not allow us to directly compare patients with eradicated and persistent SIBO in each group. We also do not monitor the presence of SIBO in patients throughout the entire observation period, and the therapy they received after the end of the treatment period. Differences in this therapy, including resolution of SIBO with antibiotic therapy due to an infectious disease, may have influenced the study results. Further studies with a larger number of patients are needed, as well as periodic analysis of SIBO in patients who have already recovered from it, to resolve the issue of the feasibility and effectiveness of repeated eradication therapy for this condition.

## 5. Conclusions

The studied probiotic containing *Saccharomyces boulardii* CNCM I-745 effectively eliminated SIBO in cirrhosis, which was accompanied by a decrease in the severity of bacterial translocation, systemic inflammation, vasodilatory endothelial dysfunction, and hyperdynamic circulation, which are the main manifestations of cirrhosis, thereby improving the medium-term prognosis for life. Considering the complexity of the cirrhosis pathogenesis and the many factors of its progression, further larger and more rigorous studies are needed to verify our data.

## Figures and Tables

**Table 1:** Main characteristics of included patients by groups.

	The Probiotic Group (*n* = 20)	The Placebo Group (*n* = 13)	*p*
Age, years	53 [46–62]	53 [45–56]	0.507
Body mass index, kg/m^2^	26.2 [23.1–29.2]	25.9 [24.0–28.4]	0.985
Male/female	6/14	6/7	0.283
Etiology of cirrhosis: alcohol	11 (55.0%)	7 (53.8%)	>0.050
Metabolism-associated fatty liver disease	2 (10.0%)	-	
HBV	1 (5.0%)	-	
HCV	3 (15.0%)	2 (15.4%)	
Mixed	2 (10.0%)	2 (15.4%)	
Cryptogenic	1 (5.0%)	2 (15.4%)	
The drugs used by the patients within the 3-month treatment period			
Beta blockers, *n* (%)	17 (85.0%)	11 (84.6%)	0.669
Spironolactone, *n* (%)	18 (90.0%)	12 (92.3%)	0.662
Loop diuretics, *n* (%)	9 (45.0%)	6 (46.2%)	0.249
Ademetionine, *n* (%)	11 (55.0%)	7 (53.8%)	0.614
Entecavir, *n* (%)	1 (5.0%)	-	0.640

**Table 2:** The changes in the main characteristics of included patients by group.

	The Probiotic Group (*n* = 20)			The Placebo Group (*n* = 13)			*p* (At Inclusion)
	At Inclusion	After Treatment	*p*	At Inclusion	After Treatment	*p*	
Child–Pugh score	8 [8–10]	7 [6–8]	<0.001	9 [8–9]	10 [9–10]	0.051	0.418
Child–Pugh class, A/B + C	0/15 + 5	8/11 + 1	0.002	0/10 + 3	0/5 + 8	1.000	1.000
End-diastolic volume of the left ventricle, mL	101 [83–115]	85 [74–100]	<0.001	95 [88–105]	100 [88–137]	0.657	0.811
Ejection fraction of the left ventricle, %	61.7 [59.7–62.7]	62.2 [60.5–63.7]	0.126	59.6 [58.0–61.0]	59.6 [57.7–63.0]	0.859	0.146
Stroke volume, mL	62 [51–72]	54 [46–62]	<0.001	57 [55–64]	63 [55–87]	0.541	0.768
Heart rate, bpm	71 [68–75]	74 [70–79]	0.087	71 [69–76]	69 [64–71]	0.422	0.581
Cardiac output, L/min	4.3 [3.5–5.1]	4.1 [3.1–4.6]	0.007	4.4 [4.0–5.2]	4.4 [3.9–6.0]	0.701	0.507
Mean blood pressure, mmHg	85 [80–90]	87 [85–93]	0.095	82 [80–89]	89 [85–98]	0.289	0.428
Systemic vascular resistance, dyn·s·cm^−5^	1513 [1281–2025]	1773 [1542–2231]	0.001	1509 [1302–1691]	1536 [1302–1816]	0.600	0.868
Systolic pulmonary artery pressure, mmHg	35 [33–39]	36 [33–40]	0.900	35 [32–37]	35 [35–37]	0.285	0.782
Nitrates, µmol/L	120 [3–160]	97 [22–169]	0.717	146 [78–174]	136 [114–150]	0.807	0.125
Claudin 3, ng/mL	12.8 [10.3–20.2]	10.4 [7.7–14.9]	0.093	15.5 [11.6–17.8]	12.1 [10.5–13.8]	0.152	0.912
Lipopolysaccharide, mEU/ml	30 [5–390]	4 [1–10]	0.010	17 [10–75]	17 [0–49]	0.155	0.971
Esophageal varices (Grade 1), *n* (%)	7 (35.0%)	6 (30.0%)	0.598	5 (38.5%)	5 (38.5%)	0.672	0.344
Esophageal varices (Grade 2-3), *n* (%)	13 (65.0%)	12 (60.0%)		5 (38.5%)	5 (38.5%)		
No esophageal varices, *n* (%)	0	2 (10.0%)	0.244	3 (23.1%)	3 (23.1%)	0.678	0.052
Minimal hepatic encephalopathy, *n* (%)	14 (70.0%)	10 (50.0%)	0.369	9 (69.2%)	10 (76.9%)	0.500	0.545
Overt hepatic encephalopathy, *n* (%)	2 (10.0%)	0		2 (15.4%)	1 (7.7%)		
Hepatic encephalopathy, *n* (%)	16 (80.0%)	10 (50.0%)	0.048	11 (84.6%)	11 (84.6%)	0.704	0.558
Ascites, *n* (%)	19 (95.0%)	8 (40.0%)	<0.001	9 (69.2%)	11 (84.6%)	0.322	0.066
Minimal ascites, *n* (%)	13 (65.0%)	6 (30.0%)	0.558	4 (30.8%)	7 (53.8%)	0.343	0.212
Clinically significant ascites, *n* (%)	6 (30.0%)	2 (10.0%)		5 (38.5%)	4 (30.8%)		
Hemoglobin, g/L	114 [105–127]	117 [100–123]	0.614	103 [88–117]	101 [84–112]	0.552	0.185
White blood cells, 10^9^ cell/L	4.4 [3.5–5.6]	4.3 [3.5–5.4]	0.614	4.0 [2.6–7.9]	3.3 [2.9–3.7]	0.117	0.839
Platelets, 10^9^ cell/L	97 [76–108]	114 [82–141]	0.001	98 [92–104]	92 [66–103]	0.017	0.912
Serum total protein, g/L	67 [61–75]	72 [70–75]	0.270	72 [67–77]	74 [61–75]	0.552	0.285
Serum albumin, g/L	33 [31–36]	38 [33–41]	0.001	33 [28–37]	31 [27–34]	0.208	0.971
Serum total bilirubin, μmol/L	36 [26–53]	28 [22–31]	0.851	57 [30–64]	60 [44–65]	0.861	0.277
International normalized ratio	1.48 [1.39–1.68]	1.49 [1.30–1.59]	0.360	1.55 [1.34–1.72]	1.61 [1.52–1.69]	0.784	0.854
Serum cholesterol, mmol/L	4.1 [3.2–5.5]	4.5 [4.1–5.0]	<0.001	3.9 [3.1–4.7]	4.3 [3.3–5.1]	0.650	0.450
Serum triglyceride, mmol/L	1.1 [0.7–1.4]	0.9 [0.7–1.2]	0.852	1.2 [0.8–1.5]	1.0 [0.7–1.2]	0.152	0.619
Serum creatinine, μmol/L	76 [62–88]	80 [72–90]	0.411	73 [68–107]	77 [71–97]	0.530	0.568
Serum sodium, mmol/L	141 [140–142]	142 [141–143]	0.025	141 [140–141]	141 [140–141]	0.965	0.811
Serum potassium, mmol/L	4.3 [4.0–4.8]	4.4 [4.1–5.0]	0.836	4.4 [4.1–4.6]	4.3 [4.1–4.7]	0.799	0.941
Serum glucose, mmol/L	4.7 [4.1–5.7]	5.2 [4.5–5.8]	0.013	4.8 [4.7–5.4]	4.8 [4.3–5.6]	0.972	0.367
Serum iron, μmol/L	14 [8–20]	15 [11–21]	0.232	9 [7–21]	15 [6–23]	0.807	0.568
Alanine aminotransferase, U/L	31 [19–43]	32 [23–39]	0.888	28 [21–51]	23 [20–50]	0.552	0.645
Aspartate aminotransferase, U/L	49 [30–67]	37 [32–46]	0.030	62 [45–80]	51 [48–72]	0.463	0.329
Gamma glutamyl transferase, U/L	61 [28–299]	56 [39–106]	0.185	98 [68–122]	116 [61–126]	0.650	0.407
Alkaline phosphatase, U/L	265 [221–372]	211 [186–259]	0.006	212 [174–287]	315 [214–359]	0.221	0.179
Cholinesterase, U/L	3596 [2875–4142]	4546 [3601–5678]	0.110	3803 [2778–5056]	3263 [2751–3851]	0.033	0.976
C-reactive protein, mg/L	8 [6–14]	7 [4–11]	0.036	7 [2–20]	7 [3–21]	0.937	0.580

**Table 3:** The changes in the main characteristics of patients in whom small intestinal bacterial overgrowth (SIBO) has been eradicated and in patients in whom it persisted.

	SIBO Eradicated (*n* = 19)			SIBO Persisted (*n* = 14)			*p* (At Inclusion)
	At Inclusion	After Treatment	*p*	At Inclusion	After Treatment	*p*	
Age, years	49 [43–62]			55 [48–57]			0.489
Body mass index, kg/m^2^	25.9 [22.6–28.7]	25.8 [24.0–29.2]	0.363	26.2 [24.0–29.0]	28.0 [24.9–29.0]	0.674	0.702
Male/female	5/14			7/7			0.151
Etiology of cirrhosis: alcohol	12 (63.2%)			6 (42.9%)			>0.050
Metabolical associated fatty liver disease	1 (5.3%)			1 (7.1%)			
HBV	1 (5.3%)			–			
HCV	2 (10.5%)			3 (21.4%)			
mixed	1 (5.3%)			3 (21.4%)			
cryptogenic	2 (10.5%)			–			
Child–Pugh score	8 [7–9]	7 [6–7]	0.008	9 [9–10]	10 [9–10]	0.374	0.131
Child–Pugh class, A/B + C	0/15 + 4	8/10 + 1	0.002	0/10 + 4	0/6 + 8	1.000	1.000
End-diastolic volume of the left ventricle, mL	94 [83–112]	88 [74–101]	0.002	98 [88–116]	99 [87–112]	0.410	0.524
Ejection fraction of the left ventricle, %	61.6 [59.6–62.7]	62.2 [60.4–63.9]	0.295	59.2 [57.9–62.1]	60.0 [58.7–63.0]	0.814	0.105
Stroke volume, mL	57 [50–69]	55 [46–62]	0.002	58 [55–7]	61 [46–67]	0.563	0.729
Heart rate, bpm	72 [67–76]	72 [68–80]	0.486	71 [68–72]	71 [68–78]	0.683	0.799
Cardiac output, L/min	4.1 [3.5–5.2]	4.0 [3.1–4.6]	0.049	4.4 [4.0–5.1]	4.2 [3.7–5.2]	0.638	0.970
Mean blood pressure, mmHg	83 [79–92]	87 [82–93]	0.408	86 [80–89]	89 [86–97]	0.090	0.855
Systemic vascular resistance, dyn·s·cm^−5^	1517 [1232–2164]	1716 [1403–2301]	0.010	1470 [1362–1798]	1812 [1478–1895]	0.140	0.870
Systolic pulmonary artery pressure, mmHg	35 [33–39]	35 [33–40]	0.944	35 [30–40]	36 [35–38]	0.398	0.985
Nitrates, µmol/L	126 [6–172]	82 [22–137]	0.420	129 [78–172]	149 [122–180]	0.198	0.608
Lipopolysaccharide, mEU/ml	35 [8–642]	4 [0–17]	0.023	17 [3–75]	4 [0–23]	0.075	0.813
Claudin 3, ng/mL	12.3 [8.5–21.2]	10.9 [8.9–15.3]	0.198	15.9 [11.9–17.8]	11.7 [8.9–13.8]	0.064	0.548
Esophageal varices (Grade 1), *n* (%)	7 (36.8%)	6 (31.6%)	0.607	5 (35.7%)	5 (35.7%)	0.515	0.430
Esophageal varices (Grade 2-3), *n* (%)	11 (52.6%)	10(52.6%)		5 (35.7%)	7 (50.0%)		
No esophageal varices, *n* (%)	1 (5.3%)	3 (15,8%)	0.302	4 (28.4%)	2 (14.2%)	0.324	0.087
Minimal hepatic encephalopathy, *n* (%)	13 (68.4%)	7 (36.8%)	0.667	10 (71.4%)	13 (92.9%)	0.269	0.269
Overt hepatic encephalopathy, *n* (%)	1 (5.3%)	0		3 (21.4%)	1 (7.1%)		
Hepatic encephalopathy, *n* (%)	14 (73.7%)	7 (36.8%)	0.024	13 (92.8%)	14 (100.0%)	0.500	0.172
Ascites, *n* (%)	17 (89.5%)	9 (47.4%)	0.006	11 (78.6%)	10 (71.4%)	0.500	0.351
Minimal ascites, *n* (%)	12 (63.2%)	7 (36.8%)	0.538	5 (35.7%)	6 (42.9%)	0.410	0.248
Clinically significant ascites, *n* (%)	5 (26.3%)	2 (10.5%)		6 (42.9%)	4 (28.6%)		
Hemoglobin, g/L	113 [97–129]	113 [96–124]	0.856	110 [88–117]	112 [92–121]	0.875	0.597
White blood cells, 10^9^ cell/L	4.5 [3.1–5.6]	4.1 [3.3–5.6]	0.857	4.1 [3.5–7.5]	3.3 [2.9–4.2]	0.101	0.841
Platelets, 10^9^ cell/L	98 [75–112]	113 [78–129]	0.043	97 [92–104]	95 [75–104]	0.851	0.757
Serum total protein, g/L	66 [61–72]	71 [69–76]	0.046	76 [69–78]	74 [70–75]	0.158	0.005
Serum albumin, g/L	35 [31–36]	37 [33–40]	0.046	32 [27–37]	31 [27–34]	0.551	0.372
Serum globulins, g/L	32 [29–38]	32 [29–38]	0.746	41 [39–55]	40 [31–48]	0.249	0.003
Serum total bilirubin, μmol/L	37 [22–61]	28 [20–35]	0.049	43 [30–64]	49 [35–62]	0.851	0.524
International normalized ratio	1.44 [1.31–1.65]	1.49 [1.26–1.64]	0.520	1.64 [1.45–1.74]	1.61 [1.46–1.69]	0.972	0.145
Serum cholesterol, mmol/L	4.1 [3.3–5.3]	4.5 [4.2–5.1]	0.444	3.7 [3.0–4.7]	4.0 [3.2–5.1]	0.363	0.183
Serum triglyceride, mmol/L	1.1 [0.7–1.5]	0.9 [0.7–1.2]	0.421	1.1 [0.8–1.4]	1.0 [0.7–1.3]	0.510	0.813
Serum creatinine, μmol/L	76 [65–107]	76 [72–96]	0.687	79 [67–103]	83 [71–97]	0.701	0.913
Serum sodium, mmol/L	141 [140–142]	142 [140–143]	0.227	141 [138–141]	141 [140–142]	0.239	0.291
Serum potassium, mmol/L	4.5 [4.0–4.8]	4.6 [4.1–5.0]	0.414	4.1 [4.0–4.5]	4.3 [4.1–4.6]	0.610	0.150
Serum glucose, mmol/L	4.9 [4.0–5.5]	4.8 [4.4–5.6]	0.240	4.8 [4.6–5.5]	5.4 [4.4–5.9]	0.300	0.649
Serum iron, μmol/L	14 [8–20]	16 [9–22]	0.421	9 [7–21]	15 [6–23]	0.551	0.771
Alanine aminotransferase, U/L	30 [18–44]	31 [18–39]	0.879	30 [21–64]	32 [20–50]	0.451	0.536
Aspartate aminotransferase, U/L	51 [31–65]	41 [36–52]	0.199	62 [37–81]	50 [30–51]	0.177	0.334
Gamma glutamyl transferase, U/L	101 [34–317]	62 [42–109]	0.116	84 [44–122]	94 [42–126]	0.470	0.649
Alkaline phosphatase, U/L	268 [227–408]	215 [187–361]	0.031	211 [174–268]	242 [201–342]	0.463	0.058
Cholinesterase, U/L	3713 [3218–4142]	4766 [3648–5678]	0.286	3358 [2778–5056]	3125 [2751–3851]	0.131	0.601
C-reactive protein, mg/L	7 [5–20]	6 [3–10]	0.033	8 [4–20]	9 [5–17]	0.972	0.827

**Table 4:** Factors of unfavorable prognosis in included patients with decompensated cirrhosis according to Cox multivariate regression analysis.

Factor	The 2 Years Follow-Up Period		The First Year Follow-Up	
	*p*	HR	*p*	HR
Hepatic encephalopathy stage *	0.001	19.9 [95% CI: 3.3–120]	0.008	16.3 [95% CI: 1.3–203]
Serum total bilirubin	0.365		0.144	
Serum albumin	0.507		0.645	
International normalized ratio	0.370		0.277	
Serum creatinine	0.173		0.116	
Ascites degree	0.450		0.374	
SIBO eradication	0.051		0.023	0.049 [95% CI: 0.003–0.657]

* 0—no hepatic encephalopathy, 1—minimal hepatic encephalopathy, 2—overt hepatic encephalopathy.

## Data Availability

Data are available upon request due to restrictions.
